# Evaluation of carcass traits, meat quality and the expression of lipid metabolism-related genes in different slaughter ages and muscles of Taihang black goats

**DOI:** 10.5713/ab.23.0418

**Published:** 2024-02-28

**Authors:** Amin Cai, Shiwei Wang, Pengtao Li, Zhaohui Yao, Gaiying Li

**Affiliations:** 1 College of Animal Science and Technology, Henan Agricultural University, Zhengzhou 10466, China

**Keywords:** Lipid Metabolism Genes, Meat Quality, Slaughter Age, Taihang Black Goat

## Abstract

**Objective:**

This study was conducted to investigate the effect of slaughter age on carcass traits, meat quality, and the relative mRNA levels of lipid metabolism-related genes in different muscles of Taihang black goats.

**Methods:**

In this study, the triceps brachii (TB), longissimus dorsi (LD) and gluteus (GL) muscles of 15 grazing Taihang black goats slaughtered at the age of 2, 3, and 4 (designated as 2-year-old, 3-year-old, and 4-year-old, respectively) were collected. The differences in carcass shape, meat quality, amino acid composition and lipid metabolism gene expression among Taihang black goats of different ages and from different plant parts were compared.

**Results:**

Compared with goats at other ages, goats slaughtered at the age of 4 had greater live and carcass weights, meat weights, bone weights and skin areas (p<0.05). LD in the 4-years-old had the lowest cooking loss and moisture content. The crude protein content in the LD of 2-year-old was significantly greater than that in the other age group, and at the age of 2, the LD had the highest crude protein content than TB and GL. The highest fat content was in LD, followed by TB, for goats slaughtered at the age of 4. Eight out of 9 essential amino acids had higher content in the TB compared with other muscles, regardless of age. The total essential amino acid content was highest in the 4-year-old and lowest in the GL muscle at the age of 3. The sterol regulatory element-binding protein-1c (*SREBP-1c*) and adipose triglyceride lipase (*ATGL*) genes were significantly more abundant in the TB muscle than in the other muscles for goats slaughtered at the age of 2. At the age of 4, the *ATGL* and peroxisome proliferator-activated receptor γ (*PPARγ*) genes were significantly more abundant in the GL than in the LD, while the fatty acid synthase (*FAS*) genes were significantly less abundant in the GL than in the other muscles. Similarly, compared with those in goats of other ages, the relative mRNA expression levels of the *FAS* and heart-type fatty acid binding protein (*H-FABP*) genes in goats slaughtered at the age of 4 were the highest, and the relative mRNA expression of the *PPARγ* gene was the lowest (p<0.05). The relative mRNA expression of the *H-FABP* and *FAS* genes was positively correlated with the intramuscular fat (IMF) content, while the relative mRNA expression levels of the *PPARγ* and *ATGL* genes was negatively correlated with the IMF content.

**Conclusion:**

Overall, a better nutritional value was obtained for TB from 4-year-old goats, in which the total essential amino acid and fat contents were greater than those of other muscles. The comprehensive action of lipid metabolism genes was consistent with that of the IMF content, among which the *FAS*, *H-FABP*, *PPARγ*, and *ATGL* genes had positive and negative effects on the process of IMF deposition in Taihang black goats.

## INTRODUCTION

The demand for mutton is increasing in China, which is particularly important for the development and utilization of local goats. The Taihang black goat, which was born and raised in Henan Province in central China, is an autochthonous local breed that is tolerant of roughage and produces high-quality meat with a low cholesterol content and high lean meat content; these products are favored by local residents. The average weight of adult Taihang black goats is 35.74 kg, and goats are covered with black or silver-black hair. However, the detailed characteristics of meat quality and lipid deposition in different slaughter ages and muscles of Taihang black goats are unclear.

As concerns about health increase, so does the demand for high-quality meat products. In recent studies, the carcass traits and meat quality of mutton have been shown to be affected by many factors, such as breed, diet and age [1,2]. With the growth of animals, the accumulation of nutrients in muscles changes. At a young age, protein accumulation is the main process involved, but after sexual maturity, the growth rate decreases. The accumulation of nutrients in muscles gradually changes from protein deposition to fat deposition.

In the improvement of meat quality, intramuscular fat (IMF) plays a crucial role. The primary components of IMF are phospholipids (found in cell membranes) and triglycerides (fat droplets), which are distributed within the epimysium, perimysium, and endomysium of muscles. Research indicates that IMF content, fatty acid composition, and various aspects of meat quality, including shear force, tenderness, flavor, and sensory scores, are closely correlated [3]. IMF weakens the connection between collagen fibers within the intramuscular connective tissue, reducing the force needed to break down the connective tissue and thereby enhancing meat tenderness [4]. Simultaneously, the lipophilic components present in IMF and their degradation products, such as aldehydes and alcohols, contribute to improving meat flavor. The deposition of fat in the body of an animal is the result of the dynamic balance between fat synthesis and breakdown metabolism. It also represents a state of equilibrium between the intake of energy substances and the organism's energy expenditure. This process is regulated by fat metabolism enzymes and various lipid-related genes, such as fatty acid synthase (*FAS*), which is the rate-limiting enzyme in the fatty acid synthesis process [5]. FAS is responsible for regulating the synthesis of fatty acids. Additionally, adipose triglyceride lipase (ATGL), a rate-limiting enzyme in the process of triglyceride hydrolysis, is significantly negatively correlated with the triglyceride content in adipose tissue [6]. Sterol regulatory element-binding protein-1c (*SREBP-1c*), peroxisome proliferator-activated receptor γ (*PPARγ*), and lipoprotein lipase (*LPL*) are important genes involved in lipid metabolism in muscle tissue [7]. Furthermore, heart-type fatty acid binding protein (*H-FABP*) participates in the transport of fatty acids [8] and is a candidate gene involved in regulating IMF deposition in goats [9].

Bharanidharan [10] reported that the age of Fabrianese lambs significantly influences meat quality. Lamb meat from older animals had significantly greater fat and protein contents. Research has shown that carcass quality was greater in goats slaughtered at the age of 2.5 than in those slaughtered at the age of 1.5 or 2. GL had a greater crude protein concentration. Amino acids and fatty acids differed among the three muscles. The expression levels of PPARγ, LPL, SREBP-1c, and ACACA were the highest in LT, followed by GL and TB, which was consistent with what has been observed for IMF content [11].

Researchers have emphasized the need to study meat quality from different breeds of goats under existing feeding systems in rural areas to establish appropriate improvement strategies for feedlotting goats [12]. As a local goat breed in Henan Province, China, the Taihang black goat has not been extensively studied for its impact on meat quality across different body parts and ages. Additionally, there is limited research on the regulatory patterns of IMF deposition genes during the growth and development of Taihang black goats. Therefore, the purpose of this study was to investigate the effects of slaughter age (the age of 2, 3, and 4) and muscle type (TB, LT, and GL) on carcass characteristics, meat quality (physical properties and chemical composition), nutritional value (amino acid content), and the expression of lipid metabolism-related genes (*PPARγ*, *SREBP-1c*, *H-FABP*, *ATGL*, and *FAS*) in three muscle types from Taihang goats across three age groups. Through these studies, our aim was to enhance the quality and nutritional value of Taihang black goat meat products, providing a scientific basis for breeding improved varieties and optimizing farming management strategies.

## MATERIALS AND METHODS

### Animals and experimental design

Fifteen healthy castrated male Taihang black goats were selected from a local grazing herd. Goats were reared under natural grazing conditions during the day and were kept overnight in pens until they were slaughtered. The goats were allowed to graze freely during the day and returned to the pen for concentrate refeeding at night until they were slaughtered. The average supplementary feed (300 g) and feed nutrient intake of the sheep are shown in Table 1. All the experimental procedures were approved by the Animal Care and Use Committee of Henan Agriculture University (Permit No. HNND2017031018) and followed the practices of the Guide for the Care and Use of Agricultural Animals outlined by the Federation of Animal Science Societies. The management procedure and diet were not different for all the goats. At this point, the goats were divided into three groups of 5 according to age (the 2-year-old, 3-year-old, and 4-year-old groups; n = 5) and slaughtered simultaneously. The average weights of the goats were 30.8 kg, 35.50 kg, and 40.10 kg for the 2-year-old, 3-year-old, and 4-year-old groups, respectively. The animals were fasted for 24 hours and subsequently transported to the local abattoir. The goats were slaughtered and cut by trained people according to the Laboratory Animal Guidelines for Ethical Review of Welfare. Approval was obtained from the ethics committee of Henan Agricultural University.

### Carcass measurements and sample collection

Before slaughter, the goats were weighed, and body weight, skin weight, bone weight, and other indicators were measured to calculate the slaughter rate and net meat percentage. Muscle samples were collected from the left half of the goat hot carcass, specifically from the triceps brachii (TB), taken from the upper arm of the trunk's forelimb, the back side of the humerus bone; longissimus dorsi (LD) at the 12th and 13th ribs, and gluteus (GL) was taken from the region at the end of the spine, below the waist and above the hips. Each sample weighed 300 grams and was stored in a cooler upon collection. After returning to the laboratory, the samples were frozen at −20°C for subsequent analysis of meat quality and nutritional value. Simultaneously, 5 to 10 grams were taken from each collected TB, LD, and GL meat sample, preserved in cryogenic tubes, and rapidly placed in liquid nitrogen. These samples were stored at −80°C in the laboratory for subsequent measurement of relative mRNA expression levels.

### Determination of physical properties and chemical composition of meat

The pH of the muscle was measured and recorded as pH_24 h_ using a portable pH meter (Testo 206; Testo International Trade (Shanghai) Co., Ltd., Shanghai, China). Muscle samples were heated in a constant-temperature water bath at 80°C for 30 minutes and cooled to room temperature. Then, the shear force was measured three times for each sample, ensuring that the muscle fibers were perpendicular to the blade, and was recorded by a C-LM muscle shear force tester (Nanjing Mingao Instrument Equipment Co., Ltd., Nanjing, China). The cooking loss and moisture and protein contents of the muscles were measured using the procedures adopted by Zhang et al [11]. The method for determining the crude fat content in muscle in accordance with GB 5009.6-2016, involved drying the muscle at 65°C, grinding it into powder, and drying the powdered sample at 105°C for 30 minutes to remove moisture, and then using the Soxhlet extraction method with organic solvent-petroleum ether for the extraction to measure the crude fat content.

### Amino acid composition

After freeze-drying and grinding, muscle samples were homogenized. An Agilent Technology HPLC (Agilent Technologies, Santa Clara, CA, USA) was used to measure the amino acid content of the muscles. Hydrolysis of the muscle, derivatization and detection of hydrolyzed amino acids were carried out following the procedure described by Zhang et al [11]. The column was composed of a reverse-phase AAA Eclipse Zorbax column (3.0×150 mm, 3.5 μm; Agilent Technologies, USA) safeguarded by an Agilent Technology precolumn.

Chromatographic separation was conducted utilizing a binary solvent system composed of two eluents: (A) 0.1 mol/L sodium acetate (CH_3_COONa) adjusted to a pH of 6.5 with a 10.0 M sodium hydroxide (NaOH) solution and acetonitrile (93:7 v/v) and (B) a mixture of acetonitrile and high-performance liquid chromatography (HPLC) grade water (80:20 v/v). The flow rate was maintained at 1 mL/min, and the elution gradient started with 0% B, which was increased to 15% B over 14 minutes, followed by a further increase to 100% B within 21 minutes, where it was sustained for 7 minutes. Subsequently, the gradient was reduced to 0% B over 3 minutes and held for an additional 15 minutes.

### Analysis of the relative mRNA levels of lipid metabolism related genes

The tissue samples, weighing 50 to 100 mg, were taken from −80°C and homogenized in 1 mL of TRIzol (Invitrogen, Carlsbad, CA, USA) using 5 mm steel beads and a multitube shaker. Total RNA extraction was subsequently performed following the instructions provided with the commercial RNA extraction kit (TaKaRa, Dalian, China). The concentration and purity of the total RNA obtained were detected using a NanoDrop One spectrophotometer (Thermo Fisher Scientific, Waltham, MA, USA). Agarose gel (1% in Tris base acetic acid ethylenediamineacetic acid [TAE] buffer; Beijing Solarbio Technology Co., Ltd., Beijing, China) electrophoresis was used to assess the integrity of the total RNA compared with molecular weight standards. Then, a TaKaRa reverse transcription kit (TaKaRa, China) was used to obtain complementary DNA (cDNA), and the specific procedure was carried out according to the instructions of the TaKaRa reverse transcription kit. According to the mRNA information of goat FAS, PPARγ, H-FABP and other lipid metabolism genes registered on the NCBI website, the primers used were designed with Primer Premier 5 software (Premier Biosoft International, Palo Alto, CA, USA) (Table 2). The primers used were synthesized by Sheng-gong Bioengineering (Shanghai, China) Co., Ltd. Real-time polymerase chain reaction (PCR) was performed using a LightCycler 96 (Roche, Basel, Switzerland) in a 20 μL reaction volume containing 10 μL of SYBR Premix Ex Taq II (2×; TaRaKa, Dalian, China), 2 μL of cDNA, 2 μL of forward and reverse primers and 6 μL of RNase-free water. The reaction process was carried out on a Light Cycler real-time quantitative PCR instrument, and the thermal cycling conditions were as follows: 5 min at 95°C for one cycle, 35 PCR cycles (30 s at 95°C, 30 s at 60°C, and 32 s at 72°C) and 10 min at 72°C, followed by melting at 95°C for 10 s and 60°C for 5 s, cooling at 50°C for 30 s, and storing at 4°C. Each sample was measured in triplicate. The expression levels of the genes were calculated by the 2^−ΔΔCt^ method [13] after the Cq value was obtained via a florescence thermal circulator (CFX96; Bio-Rad, Hercules, CA, USA).

### Statistical analysis

The data obtained were processed with Excel via WPS Office software (Jinshan Office Software, Beijing, China). The carcass trait, chemical composition and physical property, nutritional value, and lipid metabolism gene data were analyzed among slaughter ages and different muscles by one-way analysis of variance using SPSS statistics 22.0 (SPSS, Inc., Chicago, IL, USA). The results analyzed are expressed as the means and SDs. Statistical significance is defined when p-values are less than 0.05. Figures were generated with GraphPad Prism 8 software (GraphPad Software, Inc., La Jolla, CA, USA).

## RESULTS

### Carcass characteristics

The carcass characteristics are presented in Table 3. The results revealed the significant influence of slaughter age on the live weight, carcass weight, bone weight, and meat weight of the Taihang black goats (p<0.05). The 4-year-old slaughtered Taihang black goats exhibited greater live weight (40.1 kg), carcass weight (19.53 kg), meat weight (15.2 kg), and bone weight (4.17 kg) than did the 2-year-old and 3-year-old slaughtered goats (p<0.05). Additionally, the skin area and skin length of the 3-year-old and 4-year-old Taihang black goats were significantly greater than those of the 2-year-old goats (p<0.05), while no difference was observed in skin length or skin area between the 3-year-old and 4-year-old goats (p>0.05).

### Meat quality

Table 4 provides evidence of the distinct characteristics of the nutrient composition of different muscles at various ages. In 3-year-old, the TB muscle exhibited a significantly greater moisture content than did the other two age groups. Additionally, the fat content in the TB muscle was significantly lower than that in 4-year-old but did not differ significantly from that in 2-year-old. In the LD muscle, the protein content was significantly greater in 2-year-old than at the other age groups. Moreover, the highest fat content was observed at 4-year-old of LD, significantly surpassing the content at 2-year-old. No significant differences in fat content were found among the age groups in the GL. Moreover, muscle nutrients in the GL muscle were almost unaffected by age.

When analyzing the nutrient composition of muscles at different locations at the same age, a consistent pattern emerges. Regardless of age, the LD consistently exhibited the lowest moisture content. At the age of 2, the moisture content was significantly lower than that in the GL, and at the age of 3, it was significantly lower than that in the TB. Furthermore, at the age of 2, the LD had the highest crude protein content, significantly surpassing the levels in the TB and GL. Notably, there were no significant differences in crude protein content among the muscle sites for the other two age groups. At each age, the highest fat content was observed in the LD, followed by the TB, whereas it was only at the age of 4, that distinct differences in crude fat content among the different muscle sites became apparent.

### Amino acid profile

As shown in Table 5, significant differences were observed in the total content of essential and nonessential amino acids among the different muscle sites at the age of 2 (p<0.05). Differences were found in the levels of histidine, arginine, threonine, leucine, phenylalanine, tyrosine, and glutamic acid (p<0.05). At the age of 3, significant differences were observed in the essential amino acids among the three muscle sites, except for threonine (p<0.05). Furthermore, differences were found in the levels of tyrosine, alanine, glycine, and glutamic acid among the nonessential amino acids (p<0.05). Similarly, at the age of 4, significant differences were observed in the essential amino acids among the 3 muscle sites, except for tryptophan and leucine (p<0.05). Differences in the levels of tyrosine, alanine, and glutamic acid were detected for nonessential amino acids (p<0.05). The contents of methionine, isoleucine, phenylalanine, and lysine in the TB were influenced by age (p<0.05). Age also had an impact on the levels of histidine, arginine, threonine, and tyrosine in the LD (p<0.05). In the GL, the total essential amino acid content, along with phenylalanine, lysine, and cysteine content, was affected by age (p<0.05).

### The relative mRNA levels of lipid metabolism-related genes in muscles

**Figure 1:**
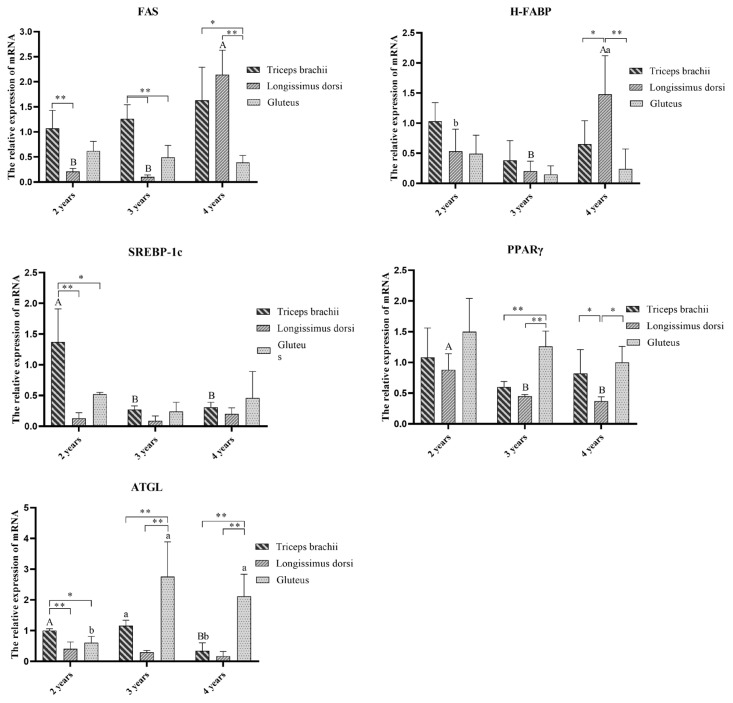
Analysis of the relative mRNA levels of lipid metabolism-related genes in muscles of Taihang black goats at different ages. Different lowercase letters indicate significant differences between slaughter age within each muscles (p<0.05), and different capital letters indicate extremely significant differences (p<0.01); * indicate significant differences between different muscles for same age (p<0.05); ** indicate extremely significant difference (p<0.01).

As shown in Figure 1, the relative mRNA levels of the *SREBP-1c* and *ATGL* genes in the GL were significantly greater at the age of 2 than at the ages of 3 and 4. Conversely, the relative mRNA levels of the *FAS* gene in the LD was notably elevated at the age of 4 when compared to that in other age groups. Moreover, the GL exhibited the highest expression of the *PPARγ* gene at the age of 2, which was significantly greater than that at the ages of 3 and 4. In the GL, the relative mRNA levels of the *ATGL* gene peaks at the age of 3, followed by the age of 4, with both age groups displaying significantly greater relative mRNA levels than at the age of 2.

Further comparison of the relative mRNA levels of lipid metabolism genes at different muscle sites at the same age revealed that at the age of 2, the relative mRNA levels of the *SREBP-1c*, *ATGL*, and *FAS* genes was highest in the TB and was significantly greater than that in the LD and GL. However, the difference between the LD and GL was not significant. Furthermore, at the ages of 3 and 4, the relative mRNA levels of the *ATGL* and *PPARγ* genes in the GL were the highest and were significantly greater than those in the other two sites. In comparison, the relative mRNA levels in these two age groups were lower than those in the TB and LD.

### Correlation analysis of the intramuscular fat content and lipid metabolism genes in the Taihang black goats

The results presented in Table 6 demonstrate a strong positive correlation between *H-FABP* and *FAS* gene expression and IMF content. Conversely, a significant negative correlation was observed between *PPARγ* and *ATGL* expression and between *PPARγ* and the IMF content.

## DISCUSSION

### Carcass characteristics

The slaughter trait of animals represents their economic value, and indices such as carcass weight, meat weight and dressing percentage indicate the level of economic benefit. Previous studies have shown that live weight and carcass weight are greater in older animals than in younger animals [11,14]. Therefore, the partial slaughter traits of Taihang black goats increase with age. The live weight of Taihang black goats slaughtered at the age of 2 was lower than that of goats (39.2 kg at the age of 2) reported by Dennis et al [15] in a grazing system. However, Hozza et al [16] reported lower live weights (mean of 18.57 kg) in crossbred goats that received different concentrate supplements and were slaughtered at the age of 21 months.

The data obtained indicated that eye muscle area and dressing percentage were not affected by slaughter age, which can be considered indicators of the degree of animal development and meat production. The dressing percentage was similar to that reported by Zhang et al [11] and Van et al [17] for goats with similar weights. Although the live weight of Taihang black goats was lower than that of Reynolds et al [18] and Sekali et al [19] (47.2 to 49.9 kg, 40.7 to 43.3 kg, respectively), the dressing percentage was higher for Taihang black goats. However, Bonvillani et al [20] reported that goats with lower live weights (10 to 13.5 kg) consumed the same amount of dressing as Taihang black goats (30 to 40 kg). These differences may be caused by differences in slaughter age, feeding system (grazing or house feeding) and breed.

### Meat quality

According to Toplu et al [21], the dry matter content of muscle gradually increases with age. However, this trend was observed only between 3-year-old and 4-year-old for each muscle in the present study. Among the different muscles, a slightly greater moisture content was found in the TB and GL than in the LD, regardless of age. The findings presented in this study agree with those of Hoffman et al [22] for Namibian gemsbok, in which the moisture content in the biceps brachii was greater than that in the LD. In contrast to our results, Kopuzlu et al [23] did not find differences in the moisture content of Eastern Anatolian Red bull muscle tissues (between LD and gluteus medius). The differences in moisture content between these studies and our study may be caused by differences in feeding type or species.

No significant differences in shear force were detected among the different muscles or slaughter ages of the goats in the present study. Tenderness is the maximum shear force perpendicular to muscle fibers when cutting meat. Brand et al [24] suggested that muscle tenderness is related to pH, sarcomere length, cooking loss and IMF. As Zhong et al [25] described, as the diameter of muscle fibers increases with age, the shear force increases gradually. In contrast, slaughter age and muscle type had no significant effect on shear force in this study, which could be attributed to the massive activity of adult Taihang black goats in the grazing system.

According to England et al [26], oxidative glycolysis of glycogen in muscle produces lactic acid, which leads to a decrease in pH after slaughter, and the glycogen decomposition ability of muscle determines the final pH and meat quality. The present study showed no statistically significant difference in the pH_24 h_ values of the muscles, but these values were within the range considered normal for animals. Similar results were reported by Abhijith et al [27], with values ranging from 5.94 to 6.01. However, the pH_24 h_ values in the muscles of the Taihang black goats in the present study were slightly greater than those described by van Wyk et al [17] for alpine rams during different periods and Oliveira et al [28] for goats older than 9 months.

Hocquette et al [29] showed that the content of IMF was positively correlated with muscle properties, including shear force, juiciness and flavor. The IMF content is affected by slaughter age and gradually increases with age [30]. The distribution of IMF content detected in the present study was similar to that reported by Kopuzlu et al [23] (GL<LD). The IMF content in the muscles of Taihang black goats slaughtered at different ages was greater than that reported by others [17]. Summermatter et al [5] reported that exercise could induce multiple adaptive effects on skeletal muscle through peroxisome proliferator-activated receptor coactivator 1 (PGC-1α) and that PGC-1α could enhance lipid oxidation, thus providing energy for sustained contraction of muscle. This finding also indicated that fat deposition in the GL of Taihang black goats was lower than that in other muscles under grazing conditions, and with increasing age and activity, there were significant differences in the IMF content among the different muscles at the age of 4.

According to Toplu et al [21], there was no significant difference in the crude protein content of Turkish wool goats slaughtered at 3 to 9 months of age, and the crude protein content was basically stable after 6 months of age. On the other hand, the crude protein content of five Spanish goat breeds with low body weights (mean of 9.5 kg) ranged from 19.52% to 24.11% [31]. Therefore, we speculate that the crude protein in the muscles of goats reaches a stable level at the age of 6 months and then does not change significantly with increasing age. The analysis above indicated that slaughter age and different muscles had small effects on the crude protein content of the Taihang black goats. However, our values were greater than those reported by Wang et al [32] for Hainan black goats slaughtered at 6 months (14.99 to 17.14 kg). These differences may be caused by differences in slaughter age and weight.

### Amino acid profile

In this study, the total nonessential amino acid content in the GL at the age of 2 and 4 was significantly greater than that in the GL at the age of 3. Additionally, the TB group contained a much greater quantity of essential amino acids than did the other muscle sites. Similar results were found in recent studies [6,33]. However, in our study, Dong et al [33] reported lower tyrosine and glutamate contents of nonessential amino acids in the TB at each slaughter age.

The content of leucine is the highest among all the essential amino acids at various muscle sites. The leucine content was the highest in previous studies reported by Zhang et al [11] and Dong et al [33], which is in line with our results. Additionally, a similar result was described by Zhou et al [34] (lysine was the highest, followed by leucine). In muscle activity, leucine can be quickly broken down and converted into glucose, which may cause a higher leucine value in the muscles of grazing Taihang black goats. For nonessential amino acids, the proline content was the highest at the age of 2 and 3, regardless of the muscle, which was different from the results of Zhang et al [11] and Wang et al [35] (the highest content of glutamate). This difference may be due to differences in breed and forage.

Regarding the effect of slaughter age on the amino acid content in muscles, the data obtained showed that the total essential amino acid and nonessential acid contents fluctuated in a V shape with age within each muscle, except for the total nonessential acid content in the LD, which increased with age. Muscles at 4-year-old showed the highest content of total essential amino acid, and lowest at 3-year-old. TB had the highest content of methionine, isoleucine and phenylalanine at 4-year-old, which were significantly different from those at 2-year-old and 3-year-old. Among all the samples, the lowest content of total essential amino acids was found in GL at 3-year-old, compared with those at 2-year-old and 4-year-old. The data obtained showed that this was caused by the low content of valine, methionine, leucine, isoleucine, phenylalanine and lysine. The distribution of total nonessential amino acids in muscle was consistent with that of total essential amino acids, except that the LD with total nonessential amino acids increased with age. The methionine content of essential amino acids and the proline content of nonessential amino acids were not significantly different in the LD at different ages, which is consistent with the description of Ilavarasan and Abraham [36].

### The relative mRNA levels of lipid metabolism-related genes in muscles

Baik et al [37] reported that the increased deposition of IMF is associated with a comprehensive effect involving elevated fatty acid production, fatty acid uptake, fatty acid esterification, and reduced lipid breakdown. The sterol regulatory element binding protein-1c (*SREBP-1c*) gene encodes SREBP-1c, which is a crucial nuclear transcription factor involved in the process of adipocyte differentiation. It participates in regulating the differentiation of adipocytes and the expression of genes related to fatty acid, triglyceride, and glucose metabolism [38]. SREBP-1c primarily regulates the lipid synthesis process in lipid metabolism. By activating a series of genes associated with fatty acid and triglyceride synthesis, such as FAS and triglyceride synthase, it promotes lipid synthesis within cells [39]. The *FAS* gene is an essential gene for fatty acid synthesis. It participates in the synthesis of intracellular fats by converting acetyl-CoA and glycerol-3-phosphate into long-chain fatty acids. The expression level of the *FAS* gene is closely linked to fat deposition. Studies have shown a significant correlation between the expression level of the *FAS* gene and the composition of fatty acids in ruminant animals [40]. Moreover, there are notable differences in the expression levels of the *FAS* gene among different muscle tissues [41]. The *ATGL* gene encodes a lipase responsible for the hydrolysis of fatty acids and is a crucial gene involved in fat metabolism. ATGLs are distributed primarily in adipose tissue, the liver, and other organs. Its primary function is to catalyze the hydrolysis of triglycerides, breaking them down into free fatty acids and glycerol. This process is a crucial step in releasing energy reserves stored in adipose tissue, providing energy for the body [42]. The activity of ATGL directly influences the rate of triglyceride breakdown in adipose tissue, thereby regulating the release of fatty acids [43]. This process is essential for maintaining overall energy balance and body metabolism. The peroxisome proliferator-activated receptor gamma (*PPARγ*) gene belongs to the peroxisome proliferator-activated receptors (PPARs) family. PPARγ is a nuclear receptor that is primarily expressed in adipose tissue, the liver, the heart, and other tissues. It plays a crucial role in regulating lipid metabolism and insulin sensitivity. PPARγ is a key regulatory factor in lipid metabolism and energy balance and participates in the modulation of fatty acid storage and breakdown. It promotes the differentiation of adipocytes and simultaneously influences insulin sensitivity [44]. The *H-FABP* gene encodes the fatty acid-binding protein, which primarily functions in binding fatty acids and participating in the regulation of lipid metabolism. It plays a role in lipid acid transport, storage, and fatty acid oxidation and is involved in the transport and regulation of goat fatty acids [45].

The gene relative mRNA levels of specific genes at different muscle locations in Taihang black goats of the same age exhibited significant variations. Specifically, at the age of 2, in the TB, the relative mRNA levels of the *SREBP-1c*, *ATGL*, and *FAS* genes was significantly greater than that in the LD and GL, with no significant difference observed between the LD and GL. The increased activity of SREBP-1c, associated with the need for increased lipid synthesis, promotes the upregulation of genes involved in fatty acid synthesis, such as *FAS*. Conversely, ATGLs primarily function in adipose tissue by hydrolyzing triglycerides to produce free fatty acids, contributing to energy provision, especially in situations requiring mobilization of lipid reserves. This may be attributed to the high forage intake and elevated lipid synthesis levels in the bodies of 2-year-old Taihang black goats during grazing, coupled with increased physical activity, resulting in higher *ATGL* gene relative mRNA levels in the TB to meet energy demands and lipid balance. At the age of 3, the relative mRNA levels of ATGL and PPARγ in the GL were significantly greater than those in the TB and LD. ATGL and PPARγ primarily participate in lipid breakdown and are negatively correlated with IMF deposition [38]. Taken together, these findings suggest that at the age of 3, the lipid metabolism activity in the GL muscle of Taihang black goats is greater than that in other locations, and IMF deposition is lower. In contrast, the TB showed the highest expression of FAS, and the relative mRNA levels of ATGL and PPARγ were significantly lower than those in the GL, indicating that the TB has a greater fatty acid synthesis capacity than the LD and GL. By the age of 4, the relative mRNA levels of FAS and H-FABP in the LD were significantly greater than those in the TB and the GL (p<0.05), while the relative mRNA levels of ATGL and PPARγ showed the opposite trend, with the lowest relative mRNA levels in the LD compared to the TB and the GL (p<0.05). This finding indicates that at the age of 4, the fatty acid synthesis capacity is greater in the LD than in the TB, and the GL exhibits the lowest fatty acid synthesis capacity. This aligns with the conclusions of a study by Zhao [46]. Therefore, as Taihang black goats age, changes in the lipid metabolism of their muscle tissues occur. At the age of 2, both lipid metabolism and synthesis rates are relatively high and are concentrated in the TB. By the age of 3, the expression of genes related to fatty acid synthesis increases in the TB, while the glutenus primarily engages in lipid metabolism. At the age of 4, the LD gradually becomes the primary site for fatty acid synthesis, and the GL exhibits the highest level of fatty acid metabolism.

The relative mRNA levels of lipid metabolism-related genes in the same muscle varies according to age. In this study, in the LD, the relative mRNA levels of the *FAS* and *H-FABP* genes showed a trend toward 4-year-old>2-year-old >3-year-old (p<0.05), while PPARγ expression was highest at 2-year-old (p<0.05). This finding suggested that in the LD, lipid synthesis is more active at 4-year-old, while lipid decomposition involving PPARγ is more active at 2-year-old. This may be because at the age of 2, goats are young and have relatively high activity levels, and the energy from feeding is mainly used for the growth of their bones and muscles. In the TB, the relative mRNA levels of the *SREBP-1c* gene were highest at 2 years old, while the expression of the *ATGL* gene was significantly greater at 2 years old compared to 4 years old. No significant differences were observed in the expression of other genes at various ages. These findings indicate that in the TB, lipid synthesis regulated by SREBP-1c is more active at 2-year-old, and this change is accompanied by greater *ATGL* gene-regulated lipid breakdown for energy release. This difference may be related to the grazing behavior of 2-year-old Taihang black goats, which involves significant foraging and increased activity. In the GL population, *ATGL* gene relative mRNA levels were highest at 3-year-old, followed by 4-year-old and 2-year-old, and there were no significant differences in the expression of other genes at different ages. These findings suggested that lipid metabolism in the GL does not exhibit a clear age-related pattern and may be related more to feeding behavior. In summary, lipid accumulation in the LD occurs mainly at 4-year-old during the later growth period in goats. In the TB, lipid synthesis is more active at 2-year-old, accompanied by lipid breakdown. However, there is no clear age-related pattern in the lipid metabolism of the GL. Gene relative mRNA levels are influenced by various factors, such as hormone levels and physical activity. Since this study lacked information on the hormone levels and activity levels of Taihang black goats at different ages, further research is needed to explore the age-related differences in gene relative mRNA levels.

### Correlation analysis of the intramuscular fat content and lipid metabolism genes in the Taihang black goats

For the relative mRNA levels of lipid metabolism-related genes in different muscles at each slaughter age, TB presented higher expression of the *SREBP-1c*, *FAS*, and *H-FABP* genes than did LD and GL when goats were slaughtered at the age of 2. Although the *ATGL* genes of the lipolysis gene had the same distribution, the distribution of IMF content in the muscles was as follows: TB>LD>GL. With respect to 3-year-old goats, the expression of the *FAS* gene was lower, and the relative mRNA levels of the *ATGL* and *PPARγ* genes was greater in GL than in the TB and LD. Similarly, at the age of 3, the expression of H-FABP and FAS was the lowest, while that of lipid decomposition genes was the highest in the GL. Fat deposition is regulated by transcription factors, and the IMF content in muscles is related to the expression level of lipid metabolism genes [11,42,44]. Finally, in our study, the comprehensive effects of fat metabolism-related genes in muscles were consistent with those of the IMF content.

## CONCLUSION

In conclusion, 4-year-old Taihang black goats can obtain greater economic benefit than 2-year-old or 3-year-old goats. The TB of 4-year-old Taihang black goats has a greater fat content and the highest total essential amino acid content, which indicates optimal nutritional value. The synthesis of lipid metabolism-related genes in the muscle of Taihang black goats was consistent with the difference in IMF content. The *FASN* and *H-FABP* genes and the *PPARγ* and *ATGL* genes had positive and negative regulatory effects on the process of IMF deposition in the muscle of Taihang black goats, respectively.

## Figures and Tables

**Table 1:** Feed composition (%)

Compositon	
Crude protein	≥15.0
Crude fiber	≤12.0
Crude ash	≤8.0
Calcium	0.7–1.2
Total phosphorus	≥0.4
Sodium chloride	0.5–1.0
Water content	≤14.0
Lysine	≥0.7

**Table 2:** The Primer sequence

Genes	GenBank ID	Sequence (5’→3’)	Product length (bp)	Amplification efficiency (%)
*β-actin*	JX046106.1	F^1)^:CGGGGAATCGTCCGTGAC	299	100.4
		R^2)^:CCGTGTTGGCGTAGAGGT		
*FAS*	NM_001314235.1	F:TCTGGGTTCACTTGTCACTGAT	217	99.8
		R:CCATCGCGTTTGCATTCACC		
*H-FABP*	XM_013971605.2	F:TGACCAAACCTACCACAA	224	102.4
		R:CACTATTTCCCGCACAAG		
*SREBP-1c*	NM_001285755.1	F:ACGGGAAAGACGACAGACAAA	147	102.7
		R:CTGACACCCCTGGAAGATGC		
*ATGL*	XM_018042656.1	F:GCGTCTACCATATCGGCGTG	670	89.2
		R:TAGCCCTGCTTGCACATCTC		

1) F, forward.

2) R, reverse.

**Table 3:** Effect of slaughter age on slaughter performance

Items	2-year-old	3-year-old	4-year-old
Live weight (kg)	30.8±1.5^c^	35.50±1.0^b^	40.10±1.60^a^
Skin length (cm)	89±13^b^	102±3^a^	106.00±4.00^a^
Skin width (cm)	71.1±4.1	76.1±8.7	80.20±4.2
Skin area (cm^2^)	6,311.3±746.6^b^	7,760.2±910.7^a^	8,477.40±376^a^
Carcass weight (kg)	14.33±1.66^c^	17.42±0.97^b^	19.53±1.41^a^
Meat weight (kg)	10.63±1.28^c^	13.47±0.95^b^	15.20±1.40^a^
Bone weight (kg)	3.53±0.48^b^	3.73±0.15^b^	4.17±0.18^a^
Eye muscle area (cm^2^)	8.94±3.39	9.34±1.82	12.42±2.18
Dressing percentage (%)	46.39±3.24	49.07±2.54	48.72±1.87
Net meat percentage (%)	34.40±2.50	37.93±2.36	37.89±2.15

a–c Different letters represent significant differences (p<0.05).

**Table 4:** Effect of slaughter age on meat quality of Taihang black goat

Items	Meat composition	2-year-old	3-year-old	4-year-old
Triceps brachii	Cooking loss (%)	39.99±2.26	36.84±4.17	37.87±1.76^AB^
	Shear force (N)	121.9±24.7	121.2±13.8	126.8±16.4
	pH_24 h_	6.0±0.1	5.9±0.1	6.0±0.1
	Moisture (%)	74.8±1.08^ABb^	75.94±0.56^Aa^	74.34±0.62^b^
	Protein (%)	21.20±0.90^B^	20.27±0.94	21.02±0.98
	Intramuscular fat (%)	3.47±0.5^ab^	3.02±0.85^b^	4.22±0.44^Aa^
Longissimus dorsi	Cooking loss (%)	39.13±2.05^a^	38.89±2.49^a^	35.56±1.45^Bb^
	Shear force (N)	129.9±24.1	137.3±49	103.60±23.80
	pH_24 h_	6.10±0.20	5.90±0.20	6.10±0.20
	Moisture (%)	73.48±2.16^B^	74.04±1.58^B^	73.11±1.75
	Protein (%)	22.86±1.32^Aa^	21.39±0.56^b^	21.25±0.93^b^
	Intramuscular fat (%)	3.03±0.59^b^	3.8±1.33^ab^	4.85±1.27^Aa^
Gluteus	Cooking loss (%)	41.75±6.65	36.92±4.61	39.91±2.17^A^
	Shear force (N)	124.7±26.7	128±26.5	123.80±34
	pH_24 h_	6.10±0.3	6.00±0.3	5.90±0.20
	Moisture (%)	75.83±0.77^A^	75.11±0.93^AB^	74.8±1.01
	Protein (%)	20.31±1.25^B^	21.16±1.13	21.72±1.03
	Intramuscular fat (%)	2.81±0.58	2.89±0.18	2.60±0.81^B^

a,b Different lowercase letters indicate significant differences between ages within the same muscles (p<0.05).

A,B Different capital letters in the same column indicate significant differences between muscles within the same ages (p<0.05).

**Table 5:** Effect of slaughter age on amino acid profile in different muscles of Taihang black goats

AA	2-year-old			3-year-old			4-year-old		
		
	TB	LD	GL	TB	LD	GL	TB	LD	GL
EAA									
His	0.36±0.09	0.42±0.20^Aa^	0.16±0.03^B^	0.35±0.05	0.22±0.05^b^	0.29±0.16	0.44±0.05^A^	0.32±0.04^B^	0.24±0.09^B^
Arg	0.96±0.09^A^	0.91±0.13^Aa^	0.23±0.10^B^	0.90±0.16^A^	0.14±0.05^Bb^	0.33±0.19^B^	1.12±0.16^A^	0.28±0.14^Bb^	0.34±0.05^B^
Thr	0.90±0.12^A^	0.62±0.09^aB^	0.55±0.14^B^	0.74±0.21	0.51±0.16^a^	0.44±0.25	0.87±0.32^A^	0.16±0.10^Bb^	0.61±0.19^A^
Val	0.36±0.03	0.31±0.06	0.30±0.07	0.35±0.05^A^	0.32±0.05^A^	0.21±0.07^Bb^	0.38±0.11	0.34±0.08	0.35±0.05^a^
Met	0.85±0.09^b^	0.71±0.17	0.75±0.07	0.81±0.11^Ab^	0.70±0.12	0.54±0.02^Bb^	0.99±0.08^Aa^	0.72±0.18^B^	0.78±0.16^a^
Iso	0.69±0.07^b^	0.56±0.14	0.56±0.07	0.67±0.10^Ab^	0.55±0.09	0.42±0.05^Bb^	0.82±0.06^Aa^	0.55±0.13^B^	0.58±0.12^aB^
Leu	1.33±0.10^A^	1.03±0.18^B^	0.97±0.23^B^	1.26±0.15^A^	1.06±0.16^A^	0.73±0.21^Bb^	1.38±0.35	1.06±0.25	1.13±0.17^a^
Phe	0.59±0.05^bA^	0.47±0.10^B^	0.50±0.03^a^	0.56±0.07^Ab^	0.48±0.07^A^	0.33±0.10^Bb^	0.68±0.06^Aa^	0.48±0.11^B^	0.51±0.08^Ba^
Lys	0.75±0.32^a^	0.84±0.08	0.82±0.08^a^	0.20±0.09^Bb^	0.69±0.19^A^	0.48±0.09^Ab^	0.24±0.09^Bb^	0.73±0.21^A^	0.93±0.15^Aa^
Total EAA	6.79±0.76^A^	5.87±0.76^a^	4.83±0.40^aB^	5.84±0.79^A^	4.68±0.84	3.77±0.70^Bb^	6.91±0.85^A^	4.65±0.84^Bb^	5.47±0.66^aB^
NEAA									
Pro	1.07±0.08	1.03±0.15	1.00±0.24	0.96±0.11^b^	1.11±0.18	1.14±0.08	1.14±0.10^a^	1.01±0.19	1.03±0.11
Tyr	0.46±0.07^C^	0.90±0.44^bB^	1.40±0.25^A^	0.45±0.08^B^	1.07±0.40^Ab^	0.83±0.39^b^	0.48±0.06^B^	1.84±0.17^Aa^	1.81±0.30^Aa^
Cys	0.20±0.04	0.16±0.04	0.21±0.00^a^	0.20±0.05	0.17±0.03	0.14±0.01^b^	0.22±0.07	0.20±0.03	0.21±0.05^a^
Ala	0.84±0.05	0.71±0.22	0.65±0.18	0.79±0.11^Ab^	0.69±0.13	0.53±0.03^B^	0.97±0.12^Aa^	0.69±0.17^B^	0.78±0.17
Gly	0.84±0.02	0.82±0.17	0.77±0.13	0.77±0.08	0.88±0.22^A^	0.57±0.16^Bb^	0.89±0.13	0.81±0.20	0.82±0.15^a^
Glu	0.34±0.05^B^	0.39±0.10	0.59±0.24^A^	0.33±0.06^B^	0.72±0.30^A^	0.47±0.18^b^	0.41±0.12^B^	0.49±0.29	0.84±0.20^Aa^
Ser	0.43±0.04	0.37±0.12	0.39±0.15	0.41±0.07	0.37±0.08	0.31±0.10	0.43±0.12	0.39±0.11	0.48±0.15
Total NEAA	4.17±0.18^B^	4.38±0.54	5.00±0.61^A^	3.92±0.45^b^	5.01±1.08	3.98±0.65^b^	4.54±0.46^aB^	5.43±0.83	5.97±0.72^Aa^
EAA/NEAA	1.63	1.34	0.97	1.49	0.93	0.95	1.52	0.86	0.92

AA, amino acid; TB, Triceps brachii; LD, Longissimus dorsi; GL, gluteus; EAA, essential amino acid; His, histidine; Arg, arginine; Thr, threonine; Val, valine; Met, methionine; Iso, isoleucine; Leu, leucine; Phe, phenylalanine; Lys, lysine; Pro, proline; Tyr, tyrosine; Cys, cystine; Ala, alanine; Gly, glycine; Glu, glutamic acid; Ser, serine.

a,b Lowercase letters are used to indicate differences between the same muscle site at different ages.

A–C Uppercase letters represent differences among different muscle sites at the same age.

**Table 6:** Analysis of the relative mRNA levels of lipid metabolism-related genes in muscles of Taihang black goats at different ages

Genes	IMF	p-values
*FAS*	0.491^**^	0.007
*H-FABP*	0.610^**^	0.000
*SREBP-1c*	0.248	0.171
*PPARγ*	−0.538^**^	0.002
*ATGL*	−0.428^*^	0.015

Significance:

* p<0.05;

** p<0.01.
